# Characterization of 1,8-diazabicyclo(5.4.0)undec-7-ene-hydroxyl-based ionic liquid for CO_2_ capture

**DOI:** 10.1098/rsos.240584

**Published:** 2025-01-08

**Authors:** Syarafana O. Amat, Normawati M. Yunus, Cecilia D. Wilfred

**Affiliations:** ^1^ Fundamental and Applied Sciences Department, Centre of Ionic Liquids, Universiti Teknologi PETRONAS, Seri Iskandar, Perak Darul Ridzuan 32610, Malaysia

**Keywords:** bistriflimide, CO_2_, DBU, dicyanamide, thiocyanate, 1,8-diazabicyclo(5.4.0)undec-7-ene

## Abstract

Six 1,8-diazabicyclo(5.4.0)undec-7-ene-based ionic liquids (ILs) linked with ethyl or propyl hydroxyl cations, coupled with thiocyanate, dicyanamide and bistriflimide anions, were synthesized through a two-step reaction: quaternization and ion exchange. The characterization of the ILs was carried out using ^1^H and ^13^C nuclear magnetic resonance spectroscopy (NMR) and Fourier-transform infrared spectroscopy (FTIR). The NMR results confirmed the structures of all the ILs, and these were supported by the FTIR results. In addition, the physicochemical properties, namely thermal stability, density and refractive index, were determined. The effects of the chain length in the cation and the identity of the anion on CO_2_ absorption were studied in a pressure drop equipment at different pressures. It was found that CO_2_ sorption increased with increasing pressure and the number of nitrile groups present. The highest CO_2_ sorption is reported to be 0.96 mol CO_2_ mol^−1^ IL at 20 bar.

## 1. Introduction

Environmental issues have been widely discussed by scientists, and the search for solvents for carbon dioxide (CO_2_) capture has become an interesting topic. Electricity generation, industrial, transportation and residential are identified as the main sectors that contribute to the high emission of CO_2_ [1]. The primary cause of anthropogenic climate change is the release of CO_2_ into the atmosphere by industrial plants [2]. This is due to rapid urbanization, which requires more consumption of energy, and about 85% of the global energy demand is met by burning fossil fuels [3]. Most industrial plants will produce large amounts of CO_2_ through combustion of fossil fuels to generate electricity. Fossil fuels are reported as the main energy resource for society with the rapid expansion of the global economy [4].

A consistent rise in CO_2_ emissions has been observed globally; for example, in China, there has been an increase from 44.3 to 128.2 million tons at an average annual growth rate of 5.2%. This increase, particularly noticeable after 2005, can be attributed to accelerated economic development [5]. Almost 30 billion tons of CO_2_ enter the atmosphere each year because of human activities [6]. The increase of CO_2_ concentrations has resulted in global warming and caused disastrous environmental consequences like increased frequency of storms, floods and droughts. It has been recorded that global sea levels have risen by 10–20 cm during the twentieth century [7]. Study by Huang & Rüther reported that global CO_2_ emissions have increased by 30% and temperature has risen by 0.3–0.68°C [8].

In the past few decades, several CO_2_ capture technologies have been investigated, including absorption, adsorption and membrane separation technologies [9,10]. Absorption separation methods can be divided into two categories: physical absorption and chemical absorption. Most commercial CO_2_ capture plants use a chemical absorption process [11]. CO_2_ removal via physical absorption is based on CO_2_ solubility in solvents. A higher CO_2_ partial pressure and a lower temperature help to raise CO_2_ solubility in the solvents. The most important and well studied solvents are selexol, rectisol and purisol [12]. The selectivity of chemical absorption is relatively high; thus, a relatively pure CO_2_ stream can be obtained. These factors make chemical absorption a suitable approach for CO_2_ capture on an industrial scale.

Amines have become one of the preferred types of solvent to capture CO_2_ owing to their ability to chemically react with CO_2_ [13]. Amines act as reactive site that can selectively bind CO_2_ molecules, facilitating their removal from gas streams. Monoethanolamine (MEA) stands out among the various amine-based solvents as a preferred solvent for capturing CO_2_. This is primarily due to its excellent CO_2_ capture capacity and rapid reaction kinetics. However, the utilization of MEA on a large scale is hindered by certain drawbacks. One such drawback is its elevated volatility, which gives rise to corrosive fumes. Consequently, these fumes pose a significant challenge in terms of process design and operation. To address this issue, the concentration of MEA needs to be restricted to a range of 15–30 wt%, resulting in added complexity and costliness in both the CO_2_ capture and solvent regeneration processes [14].

Ionic liquids (ILs) have gained significant attention as solvents in chemical processes over recent decades. Although first synthesized by Paul Walden in 1914 with ethylammonium nitrate ([EtNH_3_][NO_3_]), it was not until the 1980s that research intensified. Early applications of molten salts, such as aluminum chloride-based electroplating [15], laid the foundation for modern use of ILs. The development of stable ILs like 1-*n*-butyl-3-methylimidazolium tetrafluoroborate and hexafluorophosphate in the 1990s expanded their applications, particularly in CO_2_ capture. Room-temperature ILs (RTILs) offer unique properties, including negligible vapour pressure, low flammability and high thermal stability [16]. Ionic liquids are commonly made of an organic cation and an organic or inorganic anion [17]. The IL absorbs CO_2_ through physical interaction between its cation and anion and CO_2_. Research has shown high CO_2_ solubility in ILs like [bmim][PF_6_], along with improved absorption through amino-functionalization moieties (18,19). Functionalized ILs, such as those with amine groups or fluorine substitution, exhibit high CO_2_ absorption, making them promising candidates for gas separation and capture technologies [17]. Recent advancements in the field of ILs for CO_2_ capture have demonstrated significant progress, particularly in enhancing the efficiency and scalability of these materials. The low volatility, high thermal stability and excellent CO_2_ solubility, making them attractive alternatives to traditional solvents like amines. Key developments include the optimization of ILs through functionalization, where the anionic component plays a critical role in improving CO_2_ uptake. Functionalized ionic liquids (FUNILs), including cationic and anionic modifications, have been particularly successful in increasing CO_2_ selectivity and solubility, outperforming conventional organic solvents [20]. Research also shows that the interactions between ILs and CO_2_ molecules, especially via Lewis acid–base interactions, enhance CO_2_ absorption. Additionally, the emergence of supported ionic liquid membranes and reversible ionic liquids has provided new avenues for large-scale CO_2_ capture, even at low partial pressures [20,21]. The incorporation of molecular simulations has further supported the design of new ILs, leading to substantial improvements in their efficiency and environmental performance.

Superbase 1,8-diazabicyclo(5.4.0)undec-7-ene (DBU)-derived protic ionic liquids (PILs) are promising solvent systems for CO_2_ absorption. The absorption rate and capacity of DBU-derived PILs are in proportion to the basicity of the PIL, i.e. a more basic PIL leads to faster absorption. Previous studies have shown that coupling the DBU cation with various anions such as acetate gives capacities up to 0.8 mol CO_2_ per mole of the ILs [22] and 1.19 mol CO_2_ mol^−1^ for [DBUH][Im] [23]. Lei *et al.* reported that anions play a primary role in determining solubility of CO_2_ in ILs, while the role of cation is secondary [24]. Tokuda *et al*. reported that there is a weak acid–base interaction between CO_2_ and anion, where the anion serves as the base and CO_2_ serves as the acid [25]. Among the anions studied, we have selected bistriflamide, NTf_2_, and two nitrile base anions, thiocyanate, SCN, and dicyanamide, N(CN)_2_. Anions containing fluoroalkyl groups such as [NTf_2_] exhibited the highest CO_2_ solubility [26]. A comparison between CO_2_ sorption in ILs with fluorinated and non-fluorinated anions, namely ([emim][NTf_2_]) and ([emim][Nmes_2_]), confirmed that fluorination leads to higher CO_2_ solubility [27]. The higher CO_2_ solubility of ILs with fluorinated anions is due to the higher electronegativity of NTf_2_, leading to an increase in the strength of interaction between the anion of the IL and CO_2_ [28]. SCN and N(CN)_2_) were selected because of their potential to enhance CO_2_ capture [29]. SCN anions have attracted attention owing to the anticipated non-bonded interactions between CO_2_ and SCN [30]. Kim *et al.* reported the effect of nitrile anion in ILs on CO_2_ solubility, which suggested that ILs with a higher number of nitriles in the anion, such as C(CN)_3_ and N(CN)_2_, exhibited greater CO_2_ solubility [31].

DBU as a superbase will enhance the solubility of CO_2_. We envisage extending the absorption capability of ILs further in this research by first attaching an alcohol moiety with ethyl and propyl linker to the N atom of the imine in the DBU molecule and coupling with NTf_2_, SCN or N(CN)_2_) anion. The ILs were characterized, and their physicochemical properties such as thermal stability, refractive index (nD) and density were studied. The CO_2_ absorption studies were carried out at various pressures at 298.15 K to investigate the effect of the cation and anion for CO_2_ capture.

## 2. Material and methods

### 2.1. Materials

The main materials used in the experiment are shown in table 1. No further purification and characterization steps were performed before synthesis. All chemicals were obtained from Sigma-Aldrich.

**Table 1.** List of materials used for synthesis of the ionic liquids studied.

material	specification
1,8-diazabicyclo[5.4.0]undec-7-ene	98%
2-bromo-1-ethanol	95%
3-bromo-1-propanol	97%
sodium thiocyanate	ACS reagent, ≥98.0%
sodium dicyanamide	96%
lithium bistriflimide	99.99%
ethyl acetate	99.5%
acetonitrile	HPLC grade
CO_2_ gas	99.995%

### 2.2. Synthesis of ionic liquids

**Figure 1.**
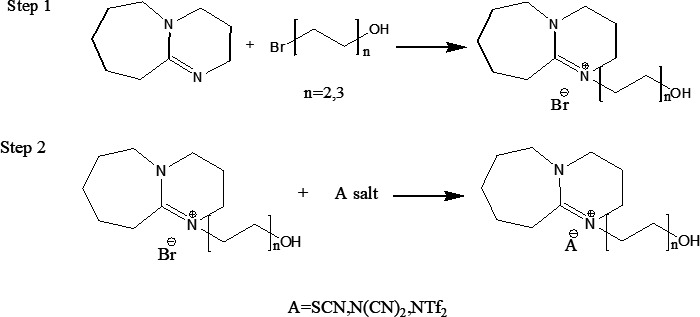
Synthesis of the ionic liquids studied.

**Figure 2.**
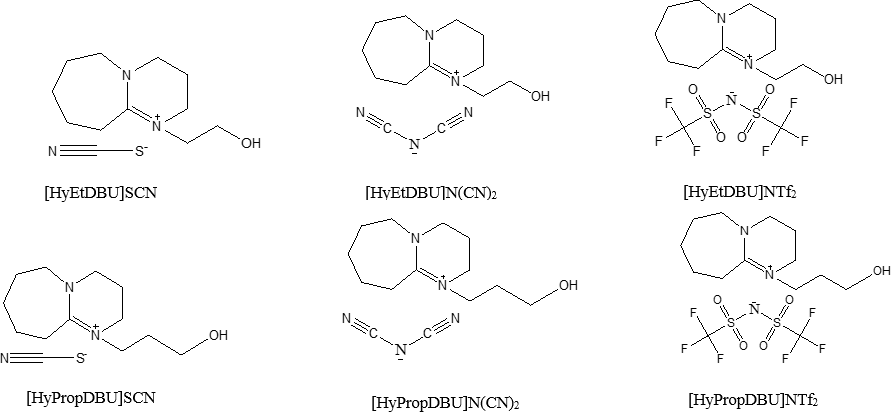
Chemical structures of and abbreviations for the synthesized ionic liquids.

The general mechanism of the synthesis is illustrated in figure 1. The structure of six DBU-based ILs linked with ethyl or propyl hydroxyl coupled with thiocyanate, dicyanamide or bistriflimide anion is shown in figure 2. DBU-hydroxyl-based ILs were synthesized through the quaternization of DBU with 2-bromoethanol in a 1 : 1 ratio in ethyl acetate at 65°C for 2 h. This process yielded the intermediate product [HyEtDBU]Br. The reaction was repeated by using 3-bromopropanol instead of bromoethanol, resulting in [HyPropDBU]Br [32]. The removal of excess ethyl acetate was carried out using a rotary evaporator, resulting in white powder ILs. Subsequently, the intermediate [HyEtDBU]Br and [HyPropDBU]Br were subjected to metathesis with sodium thiocyanate, sodium dicyanamide and lithium bistriflimide, separately in water for 3 days. [HyEtDBU]Br produced DBU-hydroxyl-based ILs with thiocyanate ([HyEtDBU]SCN), dicyanamide ([HyEtDBU]N(CN)_2_) and bistriflimide anions ([HyEtDBU]NTf_2_), respectively, while [HyPropDBU]Br gave rise to [HyPropDBU]SCN, [HyPropDBU]N(CN)_2_ and [HyPropDBU]NTf_2_, respectively. Excess water was removed using a rotary evaporator, and the ILs were washed with acetone to precipitate by-products (NaBr and LiBr). The resulting salt was filtered multiple times. Any remaining excess solvent and moisture in the ILs were removed in the rotary evaporator before characterization and application. All six DBU-hydroxyl-based ILs are liquid at room temperature.

### 2.3. Characterization

**Nuclear magnetic resonance**. ^1^H and ^13^C NMR were used for the structural confirmation using a Bruker Advance III 500 MHz instrument and dimethyl sulfoxide deuterated solvent. Chemical shifts are expressed in parts per million (ppm) with tetramethylsilane serving as the internal standard. Multiplicities are denoted as follows: s for singlet, d for doublet, t for triplet and m for multiplet. The precision of the NMR instrument is within ±0.01 ppm for chemical shift measurements, ensuring reliable identification of molecular structures.

**Water content.** The determination of water content in ILs was performed by introducing 1–1.5 g of the sample into the reagent in a volumetric Stromboli Karl Fischer oven, specifically the V30 model manufactured by Mettler Toledo. This instrument was calibrated using standard water solutions to ensure accurate titration results. The instrument’s precision is reported to be within ±0.03% of the water content.

**Fourier-transform infrared spectroscopy**. FTIR analyses were conducted using a Perkin Elmer instrument, specifically the Frontier 01 model, within the mid-infrared region of 3800–800 cm⁻¹, employing the attenuated total reflection (ATR) method. A small amount of the sample was positioned on a holder, and the presence of functional groups within the ILs was verified by identifying characteristic bands in the FTIR spectra. The FTIR instrument was calibrated using polystyrene film, which provides characteristic peaks in the mid-infrared region (3800–800 cm⁻¹). The wavenumber precision of the instrument was maintained at ±0.01 cm⁻¹, with reproducibility across three spectra measurements.

**Thermophysical properties.** Assessments of thermal stability of ILs were conducted using a thermogravimetric analyser (TGA), specifically the STA 6000 model from PerkinElmer. Approximately 5.0 mg of the sample was placed in crucible pan, which was then positioned on the sample holder. The thermal analysis was carried out from ambient temperature up to 600°C, with a heating rate of 10°C min⁻¹, under a nitrogen flow of 20 ml min⁻¹. The TGA was calibrated using standard metal weights for mass calibration and calcium oxalate for temperature calibration. The balance precision is within ±0.001 mg, and the temperature precision is ±1°C, ensuring reliable measurements of weight loss and thermal decomposition.

**Density.** Density (*ρ*) measurements for ILs were conducted utilizing a 2 ml glass pycnometer at five different temperatures (293.15, 298.15, 303.15, 308.15 and 313.15 K) under atmospheric pressure. A known quantity of the sample was introduced into the glass pycnometer, and the capillary glass stopper was securely inserted. The glass pycnometer containing the sample was then immersed in a water bath on a heating plate equipped with a thermocouple. The weight of the pycnometer was recorded, and the density was calculated using:


(2.1) $$\rho =\frac{\left(W_{IL}-W_{0}\right)}{V_{IL}},$$

where *W*_IL_ is the weight of IL with glass pycnometer, *W*_o_ is the weight of an empty pycnometer and *V*_IL_ is the known volume of IL (2 ml). This measurement process was replicated three times under the same conditions. The density measurement precision is within ±0.0001 g cm^−^³, with temperature-controlled conditions ensuring repeatability.

**Refractive index.** The nD measurements for the ILs were performed in triplicate using an ATAGO RX-5000α digital refractometer at five different temperatures (293.15, 298.15, 303.15, 308.15 and 313.15 K). A validation test was conducted using standard organic solvents supplied by the manufacturer to ensure the accuracy in sample measurement. The refractometer was calibrated using certified organic solvents provided by the manufacturer, ensuring that the instrument was within specifications. The precision of the refractometer is ±0.00002 for nD measurements, with temperature control ensuring consistent results.

### 2.4. CO_2_ absorption measurement

**Figure 3.**
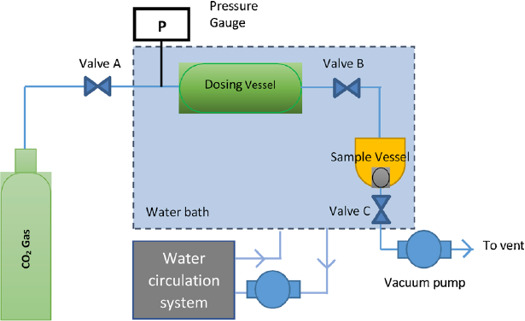
Volumetric-type high pressure manometric sorption system equipment set-up.

The study of the CO_2_ captured by the ILs was conducted using a volumetric-type high pressure manometric sorption system. CO_2_ sorption in the ILs was determined through the pressure drop method owing to the low volatility of the ILs [33]. In employing the pressure drop method, the difference in pressure within the sample cell containing the substance was recorded, and the number of moles of captured CO_2_ was calculated. The equipment set-up is presented in figure 3.

The process of capturing CO_2_ was initiated with the introduction of 0.2 ml of the ILs into the sample vessel via a micropipette. The procedure began by building up the pressure of the dosing vessel to the desired pressure. Upon achieving the desired pressure, CO_2_ gas was allowed to flow from the dosing vessel into the sample vessel. The pressure drop in the sample vessel was monitored until equilibrium was attained for both the dosing and sample vessels. The measurement of pressure drop for each gas–IL pair was conducted at room temperature (298.15 K), with pressure variations set at five different levels (1, 5, 10, 15 and 20 bar; 1 bar = 100 kPa). The calculation of the number of moles of captured gas was performed using equation (2.2):


(2.2) $$nCO_{2}=\;\frac{P_{ini}\cdot V_{tot}}{Z_{ini}\cdot R\cdot T_{ini}}-\;\frac{P_{eq}\cdot \left(V_{tot}-V_{sample}\right)}{Z_{eq}\cdot R\cdot T_{eq}},$$

where *n* is the number of moles of captured gas, *P_ini_* is the initial pressure, *P_eq_* is the pressure at equilibrium, *V_tot_* is the system volume, *V_sample_* is the sample volume, *T_ini_* is the initial temperature, *T_eq_* is the temperature at equilibrium, *R* is the gas constant (0.082057 l atm mol^−1^ K^−1^), *Z_ini_* is the compressibility factor at initial pressure and temperature, and *Z_eq_* the compressibility factor at equilibrium pressure and temperature. The compressibility factor, *Z*, was obtained by using the Peng–Robinson equation of state.

In this study, the CO_2_ captured in ILs is represented by the mole fraction ($X_{CO_{2}}$) and calculated using


(2.3) $$X_{CO_{2}}=\frac{nCO_{2}}{nCO_{2}+\;nIL},$$

where *n*CO_2_ and *n*IL represent the number of moles of CO_2_ and IL used.

## 3. Results

### 3.1. Characterization of synthesized ILs

In this subsection, the results of both ^1^H and ^13^C NMR analyses, along with the water content, for the six synthesized DBU-based ILs are provided. NMR spectra for all the ILs are shown in figures 4–18.

**Figure 4.**
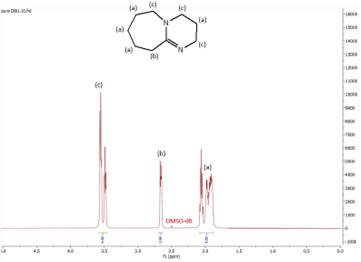
^1^H NMR specrum of pure 1,8-diazabicyclo(5.4.0)undec-7-ene (DBU).

**Figure 5.**
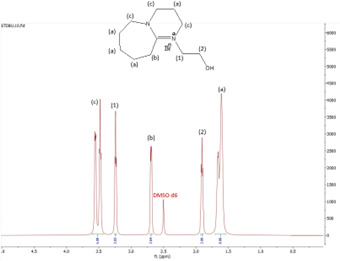
^1^H NMR spectrum of [HyEtDBU]Br.

**Figure 6.**
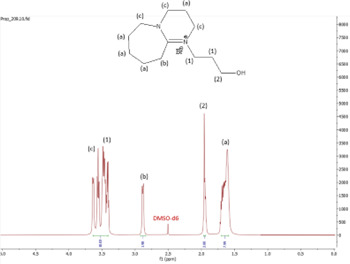
^1^H NMR spectrum of [HyPropDBU]Br.

**Figure 7.**
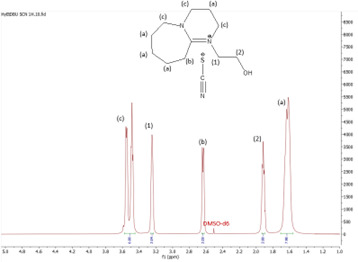
^1^H NMR spectrum of [HyEtDBU]SCN.

**Figure 8.**
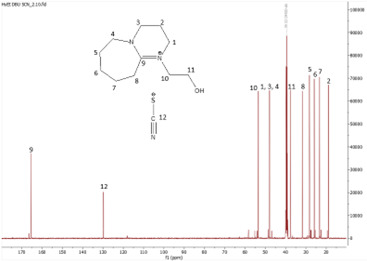
^13^C NMR spectrum of [HyEtDBU]SCN.

**Figure 9.**
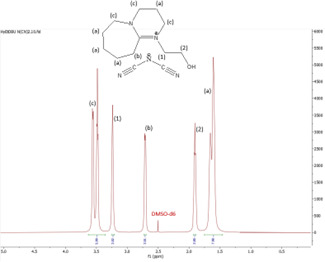
^1^H NMR spectrum of [HyEtDBU]N(CN)_2_.

**Figure 10.**
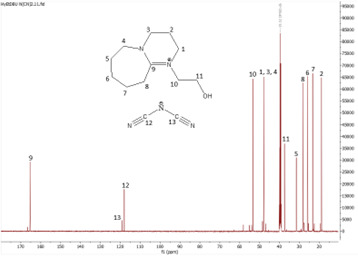
^13^C NMR spectrum of [HyEtDBU]N(CN)_2_.

**Figure 11.**
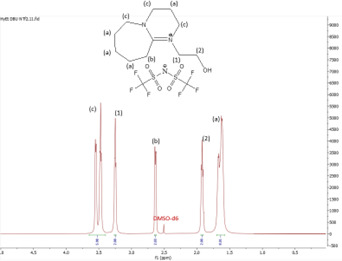
^1^H NMR spectrum of [HyEtDBU]NTf_2_.

**Figure 12.**
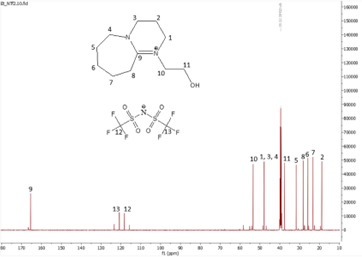
^13^C NMR spectrum of [HyEtDBU]NTf_2_.

**Figure 13.**
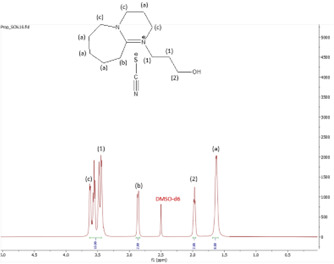
^1^H NMR spectrum of [HyPropDBU]SCN.

**Figure 14.**
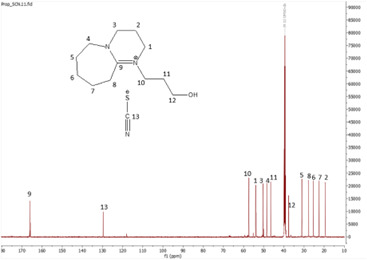
^13^C NMR spectrum of [HyPropDBU]SCN.

**Figure 15.**
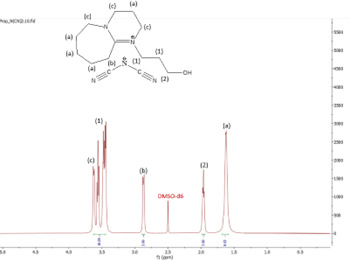
^1^H NMR spectrum of [HyPropDBU]N(CN)_2_.

**Figure 16.**
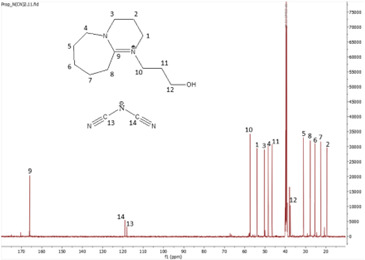
^13^C NMR spectrum of [HyPropDBU]N(CN)_2_.

**Figure 17.**
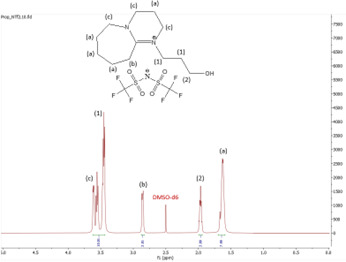
^1^H NMR spectrum of [HyPropDBU]NTf_2_.

**Figure 18.**
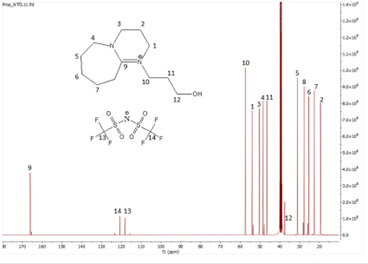
^13^C NMR spectrum of [HyPropDDBU]NTf_2_.

**[HyEtDBU][Br]:** ^1^H NMR (500 MHz, DMSO) *δ* = 3.52 (m, 6H), 2.66 (t, 2H), 1.63 (m, 8H).

**[HyEtDBU][SCN]:** ^1^H NMR (500 MHz, DMSO) *δ* = 3.52 (m, 6H), 3.24 (t, 2H), 2.63 (t, 2H), 1.91 (p, 2H), 1.62 (m, 8H). ^13^C NMR (126 MHz, DMSO) *δ* = 165.36, 129.76, 53.47, 47.93, 37.60, 31.73, 28.25, 25.90, 23.28, 18.89. Water content: 0.857%.

**[HyEtDBU][N(CN)_2_]:** ^1^H NMR (500 MHz, DMSO) *δ* = 3.52 (m, 6H), 3.24 (t, 2H), 2.71 (t, 2H), 1.91 (p, 2H), 1.62 (m, 8H). ^13^C NMR (126 MHz, DMSO) *δ* = 165.35, 119.03, 118.00, 53.38, 47.89, 37.45 (d), 31.50 (d), 28.18, 25.88, 23.28, 18.84. Water content: 1.048%.

**[HyEtDBU][NTf_2_]:** ^1^H NMR (500 MHz, DMSO) *δ* = 3.51 (m, 6H), 3.25 (t, 2H), 2.63 (t, 2H), 1.92 (p, 3H), 1.64 (m, 8H). ^13^C NMR (126 MHz, DMSO) *δ* = 165.49, 123.38, 120.83, 118.27, 115.71, 53.48, 47.92, 37.60, 31.76, 28.25, 25.89, 23.29, 18.86. Water content: 0.414%.

**[HyPropDBU][SCN]:** ^1^H NMR (500 MHz, DMSO) *δ* = 3.59 (m, 6H), 3.48 (m, 2H), 3.44 (t, 2H), 2.86 (t, 2H), 1.97 (p, 2H), 1.62 (m, 8H). ^13^C NMR (126 MHz, DMSO) *δ* = 166.13, 129.64, 57.36, 53.99 (d), 48.50 (d), 46.51, 31.07, 27.76 (d), 27.13 (m), 25.41 (d), 22.58, 19.48 (d). Water content: 1.365%.

**[HyPropDBU][N(CN)_2_]:** ^1^H NMR (500 MHz, DMSO) *δ* = 3.59 (m, 8H), 3.47 (m, 2H), 3.44 (t, 2H), 2.87 (t, 2H), 1.96 (p, 3H), 1.62 (m, 8H). ^13^C NMR (126 MHz, DMSO) *δ* = 165.95, 119.04, 118.05, 57.32, 53.92, 50.32, 48.44, 46.49, 31.05, 27.72, 27.10, 25.41, 22.58, 19.49. Water content: 2.401%.

**[HyPropDBU][NTf_2_]:** ^1^H NMR (500 MHz, DMSO) *δ* = 3.58 (m, 6H), 3.47 (m, 2H), 3.44 (t, 2H), 2.86 (t, 2H), 1.96 (p, 2H), 1.63 (m, 8H). ^13^C NMR (126 MHz, DMSO) *δ* = 166.07, 123.40, 120.84, 118.28, 115.72, 57.39, 54.00, 50.36, 48.50, 46.55, 31.07, 27.80, 27.15, 25.45, 22.61, 19.51. Water content: 0.794%.

The ^1^H NMR spectra of all the ILs exhibited a downfield shift compared with pure DBU. This shift is attributed to the introduction of electronegative groups, such as oxygen, fluorine and nitrile, during quaternization and metathesis. These groups have a highly deshielding effect, impacting the electron density around the ILs and causing a chemical shift of the cation towards the downfield region. After the quaternization step, addition of hydrogen atoms was observed. [HyEtDBU]Br displayed additional peaks compared with pure DBU, at chemical shifts of 1.91 ppm, indicating hydrogen that is attached to carbon next to the OH group, and 3.24 ppm for the hydrogen attached to carbon next to the N^+^ of the DBU cation. The chemical shift of [HyPropDBU]Br, following quaternization with 3-bromopropanol, was more deshielded compared with the ethyl chain. This is due to a higher deshielding effect caused by the longer alkyl chains. Specifically, hydrogen peaks were observed at 1.96 ppm for the hydrogen that is attached to carbon next to the OH group, at 3.45 ppm for hydrogen attached to the central carbon on the alkyl chain in the cation, and at 3.47 ppm for the hydrogen attached to carbon next to the N^+^ of the DBU cation.

The synthesis process then proceeded with the metathesis reaction whereby three anions were introduced, namely SCN, N(CN)_2_ and NTf_2_. The ^13^C NMR analysis revealed additional peaks compared with the intermediate cation after metathesis, indicating the number of carbons in the respective anions. Specifically, carbon peaks were observed at 113.3 ppm for SCN, 119.01 and 118.1 ppm for N(CN)_2_, and 120.8 and 123.4 ppm for NTf_2_. These peaks were absent in the bromide intermediates before the metathesis reaction, providing confirmation of the successful formation of the intended ILs.

The reported water content for these ILs ranges from 0.414 to 2.401%. It has been established that ILs possess highly hygroscopic properties [34]. The bistriflimide anion is known to be hydrophobic [35]; therefore, low water content was observed in the IL associated with the [NTf_2_]^−^ anion.

**Figure 19.**
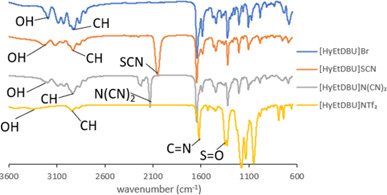
Fourier transform infrared spectra of [HyEtDBU]^+^ ionic liquids.

**Figure 20.**
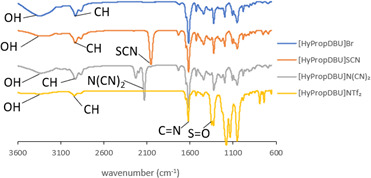
Fourier transform infrared spectra of [HyPropDBU]^+^ ionic liquids.

The structure of the ILs was further confirmed by FTIR. The FTIR spectra of the synthesized precursor of [HyEtDBU]Br and [HyPropDBU]^+^ with their corresponding anions were recorded by means of a Thermo Scientific spectrometer, using the ATR method. Figures 19 and 20 show the FTIR spectra obtained.

Compared with [HyPropDBU]Br, there are no significant differences in the absorption peaks. The presence of the strong C=N stretch observed at 1750–1500 cm^−1^ across all ILs indicated the presence of DBU. The hydroxyl stretch, typically found within the range of 3100–3700 cm^−1^, was consistently identified in all ILs. To confirm the success of the metathesis process, specific peaks were identified: the triple bond of carbon attached to nitrogen for the thiocyanate peak at 2200–2150 cm^−1^, the dicyanamide peak at 2300–2100 cm^−1^ and S=O observed at 1300 cm^−1^.

### 3.2. Thermal stability of ionic liquids

Thermal gravimetric analysis (TGA) was used to investigate the thermal decomposition characteristics of ILs. The thermograms are available in electronic supplementary material,figure S1. Examination of the TGA curves for ILs featuring [SCN]^−^ and [N(CN)_2_]^−^ anions revealed a consistent pattern characterized by two stages of degradation. The initial weight loss at a temperature below 100°C was attributed to the release of volatile components, such as absorbed moisture from the sample loading process or residual solvent from the ILs. Subsequently, the second degradation stage reflects the weight loss of the ILs and can be observed to start at temperatures exceeding 243°C for [HyPropDBU]SCN. Table 2 illustrates the thermal stability of ILs by presenting onset temperatures (*T*_o_) and decomposition temperatures (*T*_d_).

**Table 2.** The values of onset temperature (*T*_o_) and decomposition temperature (*T*_d_) for ionic liquids (ILs) studied.

IL	*T*_o_ (°C)	*T*_d_ (°C)
[HyEtDBU]SCN	264	373
[HyEtDBU]N(CN)_2_	368	416
[HyEtDBU]NTf_2_	358	494
[HyPropDBU]SCN	243	363
[HyPropDBU]N(CN)_2_	371	420
[HyPropDBU]NTf_2_	307	490

*T*_o_ represents the point of intersection between the baseline and the tangent of the sample weight plotted against temperature, while *T*_d_ indicates the temperature at which the maximum weight loss occurs [36]. ILs featuring the [N(CN)_2_]^−^ anions exhibit the highest *T*_o_, followed by those of [NTf_2_]^−^ and [SCN]^−^ anions. ILs with the [NTf_2_]^−^ anion display the highest *T*_d_, followed by [N(CN)_2_]^−^ and [SCN]^−^ anions. This observation can be attributed to the bulkier size of [NTf_2_]^−^ compared with other anions. The general trend is that thermal stability of ILs increases as the anion becomes bulkier and the negative charge is more delocalized. This finding was also observed by Chen *et al*. [34], who reported thermal stability trend anionic variations [BF_4_] < [PF_6_] < [NTf_2_]. Thermal stability increased with the number of nitrile groups in the anion; hence [N(CN)_2_]^−^ anions displayed higher thermal stability than [SCN]^−^ anions. The influence of the linker chain length between DBU and hydroxyl was not significant. Generally, *T*_d_ value decreases as we increase the number of carbons for [SCN]^−^ and [NTF_2_]^−^ anions. This is expected as longer alkyl chains contribute to increased van der Waals forces, which may reduce intramolecular electrostatic interactions, resulting in lower thermal stability [37]. However, this was not observed with [N(CN)_2_]^−^ anions.

### 3.3. Density thermal expansion coefficient, standard entropy and lattice potential energy measurements

**Figure 21.**
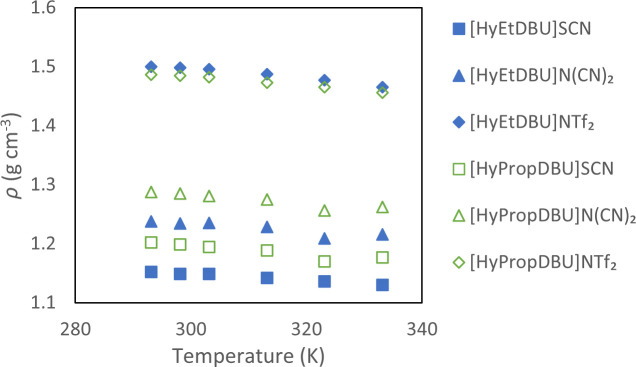
Density of the ionic liquids at 293.15−333.15 K.

The investigation of the ILs' density was carried out over a temperature range from 293.15 to 333.15 K. The plots of the experimental density of the ILs are shown in figure 21, while the detailed data are accessible in electronic supplementary material, table S1. The graphical representation in figure 21 reveals a consistent linear decrease in the densities of all six ILs with increasing temperature.

Elevated temperatures lead to an expansion in the volume of ILs, resulting in a decreasing density. At higher temperatures, weakened intermolecular forces between constituent ions enhance ion mobility, contributing to increased volume [38]. The experimental findings suggest that the ILs' density decreased with bigger NTF_2_ anions when the linker chain length increased in the cations. This is due to increased volume occupied by the longer chains, resulting in reduction of the overall density of the IL [39]. For smaller anions such as SCN and N(CN)_2_, the increase in the linker length on the cation side contributed to closer packing of the ILs, which led to reduced volume and therefore increased density.

The thermal expansion coefficient (*α*) was computed using the equation:

**Table 3.** Thermal expansion coefficients (*α*_*p*_) of the ionic liquids studied.

*T* (k^−1^)	10^−4^ *α* (K^−1^)					
	[HyEtDBU]SCN	[HyEtDBU]N(CN)_2_	[HyEtDBU]NTf_2_	[HyPropDBU]SCN	[HyPropDBU]N(CN)_2_	[HyPropDBU]NTf_2_
293.15	4.79	5.58	5.96	6.49	6.01	5.40
298.15	4.80	5.57	5.89	6.46	5.99	5.34
303.15	4.73	5.46	5.85	6.47	5.98	5.31
313.15	4.75	5.47	5.86	6.43	5.96	5.34
323.15	4.77	5.78	5.87	6.72	6.23	5.34
333.15	4.79	5.44	5.94	6.35	5.88	5.37


(3.1) $$\alpha_{p}=-1/\rho \;\times \left(\frac{\delta \rho }{\delta T}\right),$$

where *p* represents constant pressure. The corresponding data are organized in table 3. Generally, *α* represents the expansion amount of substance in response to a change in temperature [40]. In contrast to prior research where *α* exhibited an increase with increasing temperature, the data in table 3 reveal negligible variations in *α* for this study. This suggests that the thermal expansion coefficient of the ILs remains unaffected by changes in temperature. The calculated values of *α* for all ILs were in the range of 4.73 × 10^−4^ to 6.72 × 10^−4^, which is lower than for common solvents such as acetone (1.10 × 10^−3^ K^−1^), chloroform (1.27 × 10^−3^ K^−1^) and ethyl acetate (1.38 × 10^−3^ K^−1^; [41]).

The calculated values presented in table 3 demonstrate a slight variation in the thermal expansion coefficient with an increase in the carbon numbers within the structure of the ILs. ILs with [HyPropDBU]^+^ exhibit a higher *α*_*p*_ compared with those with the [HyEtDBU]^+^ cation. This suggests that the thermal expansion coefficient is influenced not only by cation symmetry but also by the linker’s chain length [42]. The thermal expansion coefficient behaves similarly for all ILs, displaying results that can be considered as temperature-independent, given the consistent outcomes across the studied temperature range. Similar trends in the variation of the thermal expansion coefficient have been reported by Sarkar *et al*. for diethylammonium-based ILs [43].

Molar volume, *V*_m_, is the space occupied by one mole of a substance at a specific temperature and pressure. The computation of the molar volume involved the application of an empirical equation utilizing experimental density data [44]:


(3.2) $$V_{m}=\frac{M}{\rho },$$

where *M* (g mol^−1^) is the molecular weight and *ρ* (g cm^−3^) the density. The calculated *V_m_* values are presented in table 4. ILs with [NTf_2_]^−^ occupy more volume when compared with [SCN]^−^ and [N(CN)_2_]^−^ anions owing to the increase in the size of the ILs. Similarly, the [HyPropDBU]^+^ cation occupies more volume as compared with [HyEtDBU]^+^ with the same anions. [HyPropDBU]NTf_2_ has the largest molar volume, owing to weak molecular forces resulting from the bulky size and unsymmetrical arrangement [45].

**Table 4.** The values of molar mass, molecular mass (*V*_m_), molar volume (*V*), standard entropy (*S*°) and lattice potential energy (*U*_pot_) or the studied ionic liquids (ILs) at various temperatures (*T*).

IL and *T* (K)	molar mass (g mol^−1^)	*V*_m_ × 10^2^	*V* (nm^3^)	*S֯*° (J K^−1^ mol^−1^)	*U*_pot_
[HyEtDBU]SCN	272.3664				
293.15		2.36	0.4226	556.3	424.2
298.15		2.37	0.4310	566.7	423.9
303.15		2.37	0.4384	575.9	423.9
313.15		2.38	0.4553	597.0	423.3
323.15		2.40	0.4723	618.2	422.7
323.15		2.41	0.4896	639.7	422.1
[HyEtDBU]N(CN)_2_	280.3285				
293.15		2.27	0.3934	519.9	428.8
298.15		2.27	0.4011	529.5	428.5
303.15		2.27	0.4076	537.6	428.6
313.15		2.28	0.4235	557.4	428.0
323.15		2.32	0.4438	582.7	426.3
333.15		2.31	0.4550	596.7	426.9
[HyEtDBU]NTf_2_	494.4264				
293.15		3.30	0.3245	434.0	390.6
298.15		3.30	0.3304	441.4	390.5
303.15		3.31	0.3365	449.0	390.3
313.15		3.33	0.3498	465.5	389.8
323.15		3.35	0.3632	482.2	389.2
323.15		3.37	0.3775	500.1	388.4
[HyPropDBU]SCN	286.3914				
293.15		2.38	0.4050	534.3	423.4
298.15		2.39	0.4129	544.2	423.1
303.15		2.40	0.4213	554.7	422.7
313.15		2.41	0.4375	574.8	422.2
323.15		2.45	0.4586	601.2	420.5
333.15		2.4	0.4702	615.6	421.1
[HyPropDBU]N(CN)_2_	294.3535				
293.15		2.29	0.3781	500.7	427.8
298.15		2.29	0.3854	509.9	427.6
303.15		2.30	0.3929	519.3	427.3
313.15		2.31	0.4080	538.1	426.7
323.15		2.34	0.4273	562.1	425.1
333.15		2.33	0.4382	575.7	425.7
[HyPropDBU]NTf_2_	508.4514				
293.15		3.42	0.3276	437.8	387.1
298.15		3.42	0.3334	445.1	387.0
303.15		3.43	0.3397	452.9	386.8
313.15		3.45	0.3531	469.6	386.2
323.15		3.47	0.3663	486.1	385.7
333.15		3.49	0.3800	503.2	385.1

Molecular volume (*V*) can be estimated from:


(3.3) $$V=\frac{V_{m}}{N_{A}},$$

where *V*_m_ is molar volume and *N*_A_ is the Avogadro constant. The calculated molecular volumes of the ILs are listed in table 4; they follow a similar trend to that expected for the molar volumes of the ILs. The standard molar entropy (*S֯*°) and lattice potential energies (*U*_pot_) of the ILs were calculated from molecular volume by using the equations developed by Glasser [46]:


(3.4) $$S^{\circ }=1246.5\;\times V\;\left(nm^{3}\right)+29.5,$$

(3.5) $$U_{pot}=ϒ\;(\rho /M{)}^{\frac{1}{3}}+\delta ,$$

where *ϒ* and *δ* are fitting coefficients with values of 1981.9 kJ mol^−1^ and 103.8 kJ mol^−1^, respectively.

The lattice potential energy of the ILs was calculated for a temperature range from 293.15 to 333.15 K. The primary factor that affects lattice potential energy is the electrostatic or Coulombic interaction. However, lattice potential energy exhibits an inverse correlation with ion volume [47]. Table 4 illustrates that lattice potential energy experiences a decrease when the linker chain length of the cations is increased. The introduction of a methylene group (-CH_2_-) into the cation’s alkyl chain enhances entropy, consequently decreasing packing efficiency in the ILs [48].

### 3.4. Refractive index

**Figure 22.**
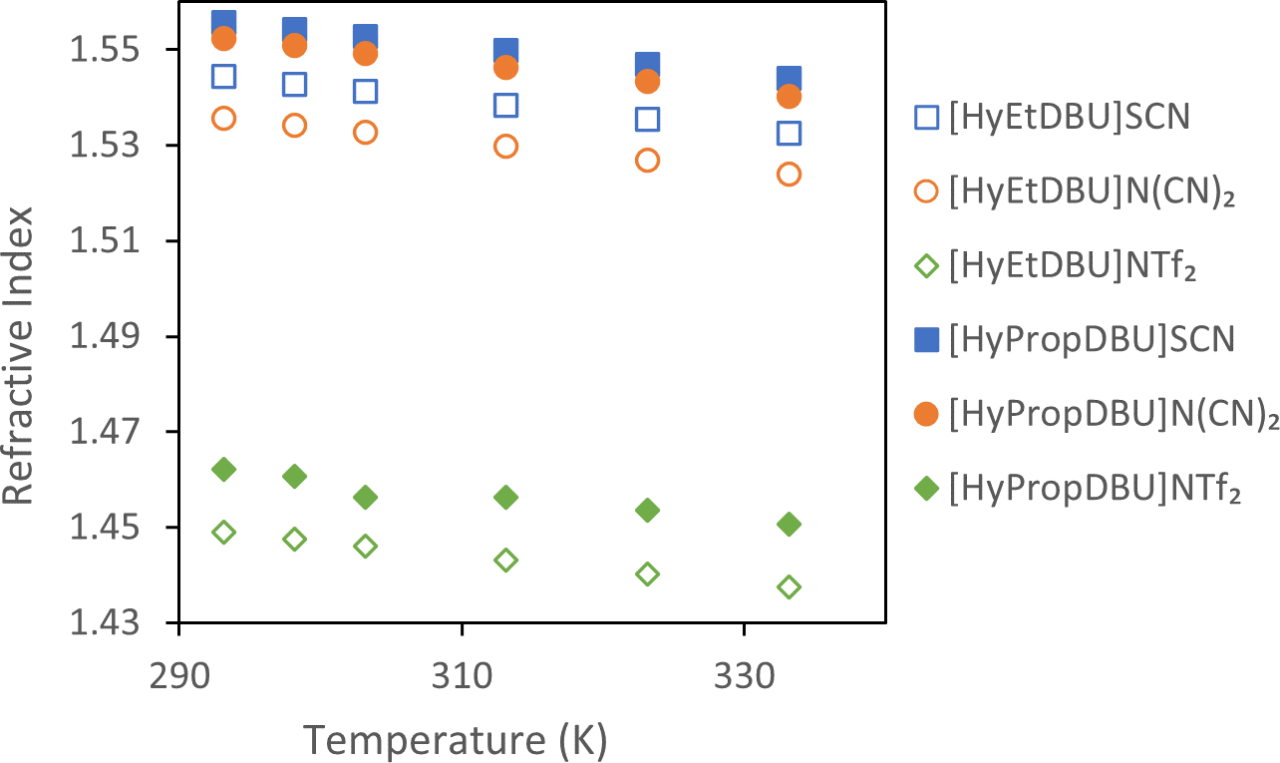
Graph of refractive index against temperature for the ionic liquids studied.

Refractive index (nD) can be defined as the speed of light propagation through a material, providing an estimate of the electronic polarizability of molecules and indicating the dielectric response to an external electric field generated by electromagnetic waves [49]. Figure 22 depicts the nD of ILs measured at 293.15 to 333.15 K at atmospheric pressure. The experimental data can be found in electronic supplementary material, table S2. It was observed that the nD values exhibited a decrease as the temperature increased. This is due to the increased molecular motion and reduced density of the liquid. The nD values tend to increase with the increase of the linker chain length of the DBU-hydroxyl cations. This rise in nD values could be attributed to enhanced intermolecular interactions, particularly the van der Waals forces among the ILs [47].

The nD value can be utilized to determine the electronic polarizability or molar refraction (*R*_m_) using the Lorentz–Lorenz relation, as expressed in equations (3.6) and (3.7). The calculated *R*_m_ value can then be employed to derive the free volume (*V*_f_) of the ILs. Free volume refers to the unoccupied space between molecules, arising from the static and dynamic disorder of the chemical structure [50]. This parameter is important for understanding the solubility of gases in ILs. The calculation of *R*_m_ and *V*_f_ can be performed using the following equations:


(3.6) $$R_{m}=\left(\frac{RI^{2}-1}{RI^{2}+2}\right)V_{m},$$

(3.7) $$V_{f}=V_{m}-R_{m},$$

where *R*_m_ is molar refraction, nD is the refractive index, *V*_m_ is the molar volume of the ILs, and *V*_f_ is the free volume of the ILs.

As presented in table 5, the values of *R*_m_ do not exhibit a consistent trend with increasing temperature. Thus it can be said that *R*_m_ value is not dependent on temperature. This finding aligns with the results obtained by Zhang *et al.* in their investigation of ether FUNILs combined with amino acid anions, where they attributed this behaviour to the induced dipole effect of the ILs [51]. Table 5 also illustrates that *V*_f_ is directly proportional to the molar mass of the ILs and increases with increasing temperature. In line with this, the [HyEtDBU]^+^ cation, with the same anions, possesses a lower molar mass and consequently a lower *V*_f_ value when compared with the ILs with [HyPropDBU]^+^ as the cation. This can be attributed to the smaller size of the [HyEtDBU]^+^ cation compared with [HyPropDBU]^+^.

**Table 5.** The values of the square of refractive index (nD^2^), molar refraction (*R*_m_) and free volume (*V*_f_) with temperature (*T*) for the ionic liquids (ILs) studied.

IL	*T* (K)	nD^2^	*R*_m_	*V*_f_
[HyEtDBU]SCN	293.15	2.40982	75.60	160.9
	298.15	2.40507	75.63	161.5
	303.15	2.40036	75.48	161.7
	313.15	2.39098	75.55	162.9
	323.15	2.38177	75.59	164.1
	333.15	2.37249	75.66	165.4
[HyEtDBU]N(CN)_2_	293.15	2.40982	72.43	154.1
	298.15	2.40507	72.44	154.7
	303.15	2.40036	72.24	154.8
	313.15	2.39098	72.32	156.0
	323.15	2.38177	73.11	158.7
	333.15	2.37249	72.38	158.2
[HyEtDBU]NTf_2_	293.15	2.40982	88.42	241.2
	298.15	2.40507	88.26	241.7
	303.15	2.40036	88.16	242.4
	313.15	2.39098	88.20	244.4
	323.15	2.38177	88.25	246.4
	333.15	2.37249	88.47	248.9
[HyPropDBU]SCN	293.15	2.42026	76.55	161.7
	298.15	2.41554	76.58	162.3
	303.15	2.41094	76.67	163.0
	313.15	2.40169	76.72	164.2
	323.15	2.39271	77.61	167.2
	333.15	2.38381	76.84	166.6
[HyPropDBU]N(CN)_2_	293.15	2.35847	71.25	157.4
	298.15	2.35386	71.26	157.9
	303.15	2.34929	71.28	158.5
	313.15	2.34014	71.31	159.6
	323.15	2.33106	72.03	162.4
	333.15	2.32221	71.33	161.8
[HyPropDBU]NTf_2_	293.15	2.13771	94.08	248.1
	298.15	2.13353	93.91	248.5
	303.15	2.12107	93.33	249.8
	313.15	2.12107	93.91	251.3
	323.15	2.11287	93.93	253.2
	333.15	2.10468	94.00	255.3

### 3.5. CO_2_ absorption

**Figure 23.**
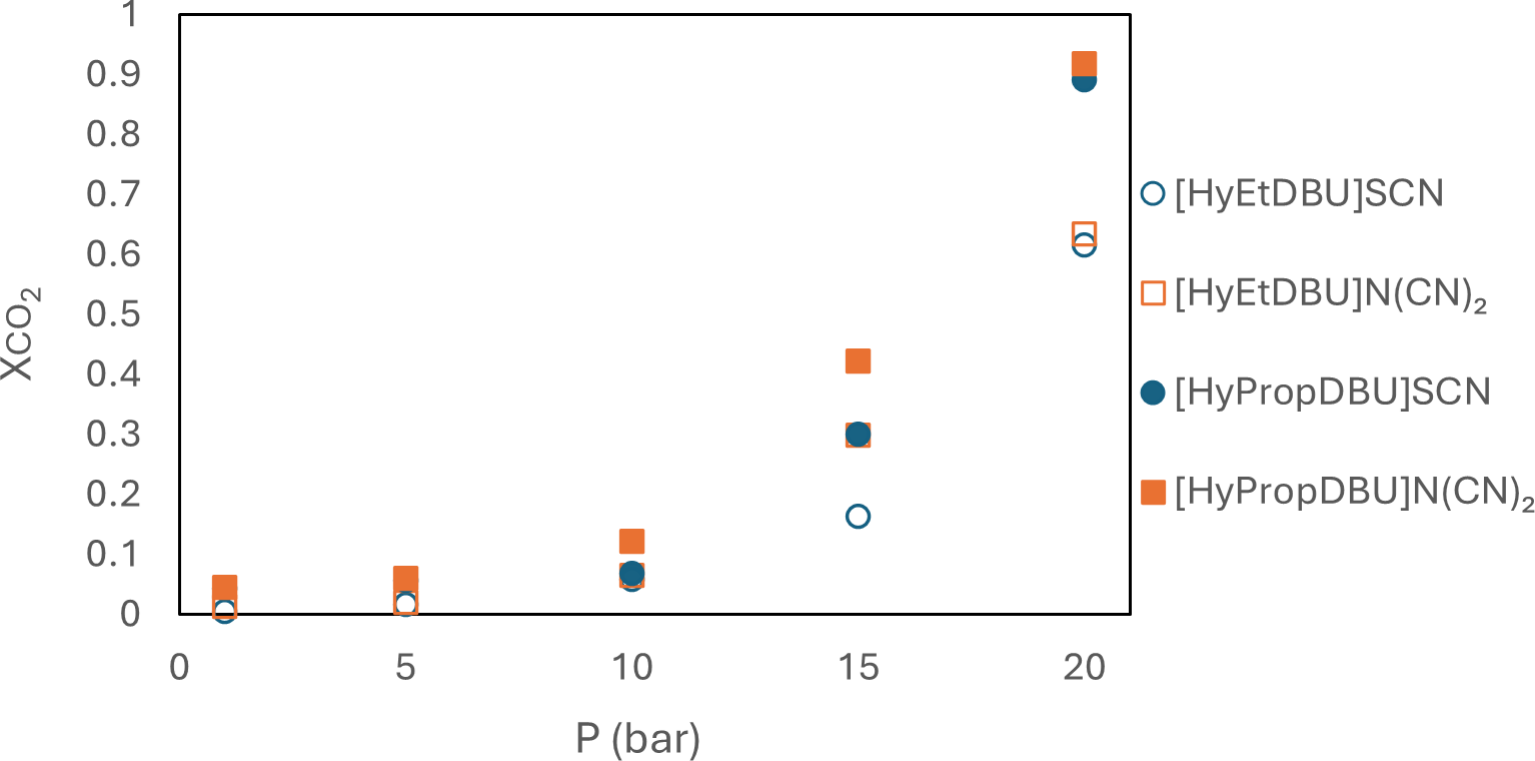
CO_2_ absorption of the studied ionic liquids with nitrile anions.

In this study, the performance of ILs with different nitrile anions and different alkyl chains in capturing CO_2_ was evaluated in terms of mole fraction, *X*CO2; the findings are presented in figure 23. The results indicated that, at 298.15 K and pressures of 1, 5, 10, 15 and 20 bar, the mole fraction of CO_2_ absorbed by the ILs ranged from 0.005 to 0.92 for the nitrile anions. The performance of the ILs in CO_2_ absorption follows the sequence [HyPropDBU]N(CN)_2_ > [HyPropDBU]SCN > [HyEtDBU]N(CN)_2_ > [HyEtDBU]SCN. ILs with the [HyPropDBU]^+^ cation exhibited the highest CO_2_ absorption, specifically 0.89 and 0.91 for [HyPropDBU]SCN and [HyPropDBU]N(CN)_2_, respectively. The ILs with the N(CN)_2_ anion generally demonstrated greater CO_2_ absorption compared with SCN^−^ anions. Kim *et al*. previously reported that the dissolved CO_2_ mole fraction was 0.6 and 0.5 for [c2mim]N(CN)_2_ and [c2mim]SCN, respectively, at 7 bar and 60°C [30]. The interaction between CO_2_ and the IL involves a Lewis acid–base mechanism, where CO_2_ acts as a Lewis acid and the nitrile anion in the IL acts as a Lewis base [52].

**Figure 24.**
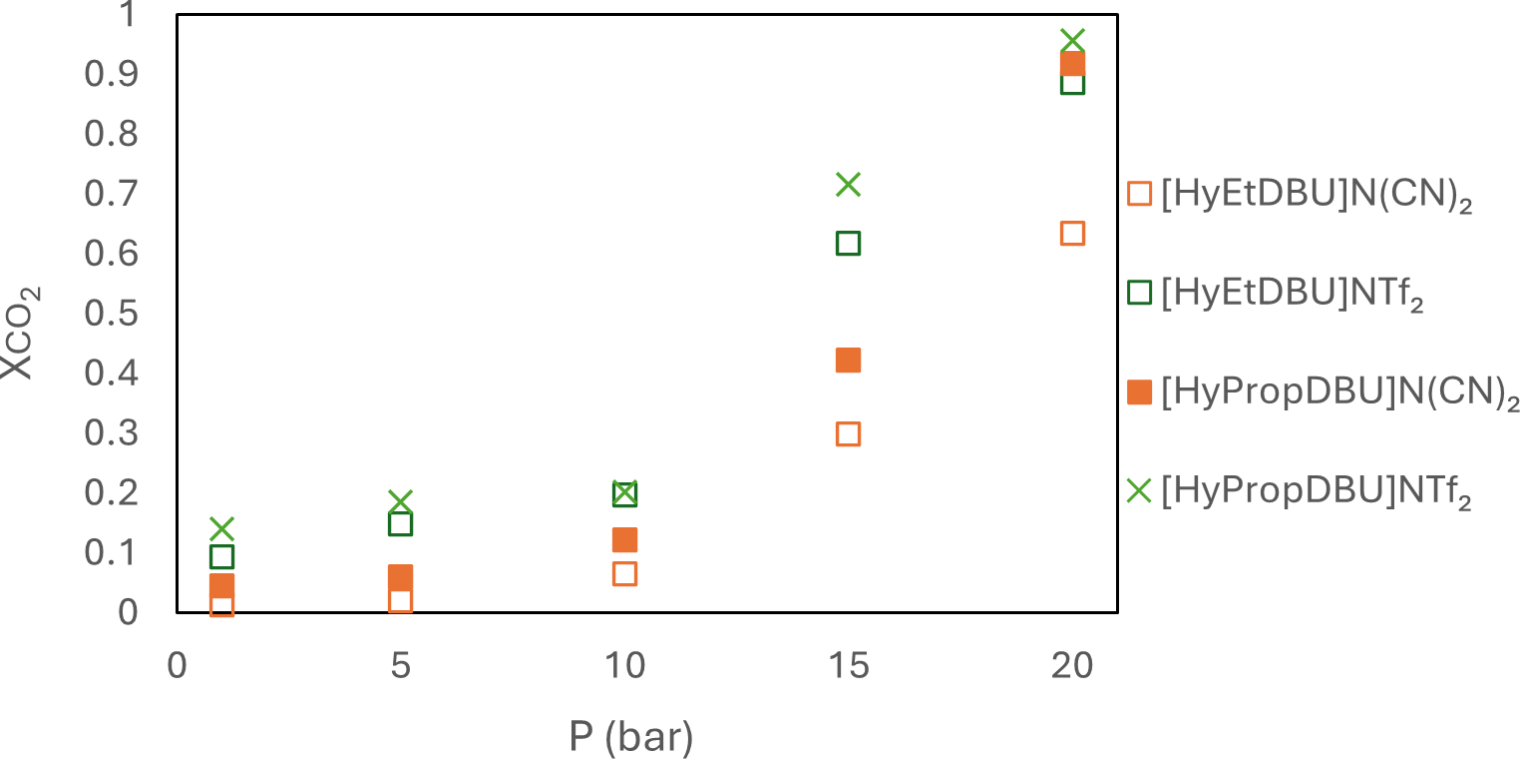
CO_2_ absorption of the ionic liquids with [NTf_2_]^−^ and [N(CN)_2_]^−^ anions.

A study conducted by Liu *et al.* established a significant linear relationship between the solubility of CO_2_ in ILs and the fractional *V*_f_ value [53]. According to our experimental findings, an increase in the molar mass of ILs corresponded to an increase in the *V*_f_ value and, consequently, an enhancement in the ILs' capacity to absorb CO_2_. Figure 24 shows an increase in CO_2_ absorption when the anion was changed from [N(CN)_2_]^−^ to [NTf_2_]^−^ and when the linker chain length on the cations increased from *n* = 2 to 3. At 20 bar, the mole fraction of CO_2_ absorbed by [HyPropDBU]N(CN)_2_ and [HyPropDBU]NTf_2_ was 0.92 and 0.96, repectively. [HyPropDBU]NTf_2_ has the largest *V*_f_ value in this study, which can be related to having more space for gas absorption, hence overall improvement in the CO_2_ solubility of the ILs. In addition, the [NTf_2_]^−^ anion contains two fluoroalkyl groups, which are known to increase CO_2_ solubility.

When compared with other DBU ionic liquids (table 6), NTf_2_, N(CN)_2_ and SCN anions showed similar CO_2_ absorption to those studied by Gao *et al.* [23] and Losetty *et al.* [22]. The anions used in the current work are weaker bases compared with imidazolate, pyrazole and 4-methoxyphenolate; hence, their rate of CO_2_ absorption is slower than for those more basic anions. Our work proves that coupling DBU-hydroxyl as the cation with NTfs and nitrile-containing anions also significantly influences the performance in CO_2_ capture processes. In addition, the length of the linker chain length attached to the DBU cations can alter the CO_2_ uptake capabilities. All six ILs in this work exhibited high thermal decomposition temperatures (>350℃). A higher CO_2_ absorption is achieved when operating at higher pressure. There is no reported work on the performance of [DBUH]imidazolate PILs at high pressures specifically for CO_2_ capture applications. Our results have shown that the ILs from this work could be used at high temperature and pressure, which gives them the potential to be used in post-combustion CO_2_ capture. Potentially, these ILs could be integrated with porous materials to create solid-supported ILs. These hybrid materials would combine the advantages of the ILs' selectivity and low vapour pressure with the high surface area of porous supports, making them effective for CO_2_ separation from flue gases and natural gas.

**Table 6.** CO_2_ absorption data for a range of 1,8-diazabicyclo(5.4.0)undec-7-ene (DBU) ionic liquids (IL).

IL	mole fraction, *X*CO_2_	operating conditions
[DBUH][Im]	1.19	25^ο^C, 1 bar [23]
[DBUH][Pyra]	1.15	
[DBUH][4-MP]	0.90	
[DBU]Ac	0.7992	30^ο^C, 20 bar [22]
[DBU]TFAc	0.5154	
[DBU]TFMSA	0.4937	
[DBUMSA]	0.4026	
[HyEtDBU]SCN	0.6161	25^ο^C, 20 bar (this work)
[HyPropDBU]SCN	0.8908	
[HyEtDBU]N(CN)_2_	0.6342	
[HyPropDBU]N(CN)_2_	0.9191	
[HyEtDBU]NTf_2_	0.8863	
[HyPropDBU]NTf_2_	0.9573	

### 3.6. Henry’s Law constant

**Figure 25.**
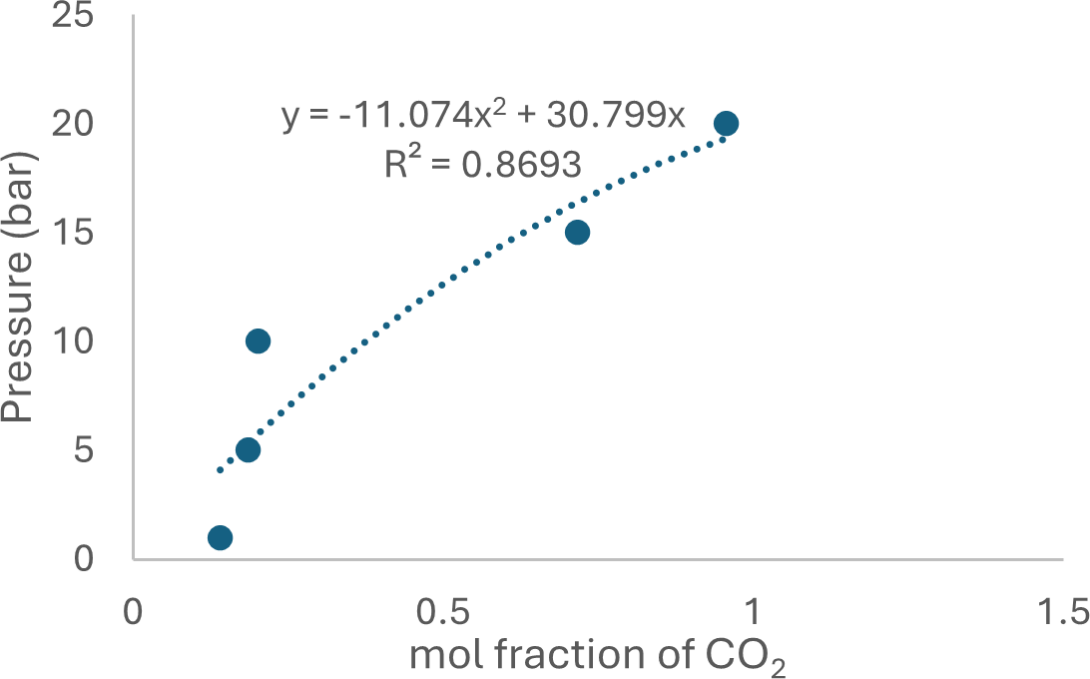
The second-order polynomial of [HyPropDBU]NTf_2_.

Henry’s Law constant, *K*_H_ , is commonly employed to describe the solubility of gases in liquids. Estimation of *K*_H_ value involves the use of solubility expressed in terms of mole fraction (*X*CO_2_). In the case of gases behaving almost ideally, such as C_2_H_4_, C_2_H_6_, CH_4_, O_2_ and Ar, solubility is linearly correlated with pressure, allowing the *K*_H_ value to be determined by calculating the linear slope of the data. However, for CO_2_, a deviation from linearity is observed as the CO_2_ pressure gradually increases, as illustrated in figure 25. Consequently, to determine the *K*_H_ value for CO_2_, a second-order polynomial is fitted to the data, and the limiting slope is calculated as the solubility approaches zero [54]. The experimental values were modelled using:

**Table 7.** Henry’s constant (*K*_H_) and correlation coefficient (*R*^2^) for the ionic liquids (ILs) studied.

IL	*K*_H_	*R* ^2^
[HyEtDBU]SCN	127.51	0.95
[HyPropDBU]SCN	71.84	0.87
[HyEtDBU]N(CN)_2_	81.92	0.90
[HyPropDBU]N(CN)_2_	55.83	0.92
[HyEtDBU]NTf_2_	37.62	0.92
[HyPropDBU]NTf_2_	30.80	0.87


(3.8) $$K_{H}=ax^{2}+bx+c,$$

resulting in a correlation coefficient (*R*_2_) greater than 0.87. The corresponding data are presented in table 7. The graph of the second-order polynomial for [HyPropDBU]NTf_2_ is plotted in figure 25. Graphs of the second-order polynomial for the other ILs are available in the electronic supplementary material, figures S2−S7.

The solubility of a gas can be represented by the *K*_H_ value, which establishes a relationship between the mole fraction of a substance in the liquid phase and its partial pressure in the gaseous phase. A descending order of *K*_H_ values signifies an increase in the degree of gas solubility in ILs. The trend in table 7 shows that for ILs having the same anion, adding one carbon to the linker length of the cation results in a decrease in *K*_H_ value, indicating enhanced CO_2_ sorption. For instance, [HyEtDBU]SCN has a *K*_H_ value of 127.51, whereas [HyPropDBU]SCN exhibits a *K*_H_ value of 71.84. Similarly, [HyEtDBU]N(CN)_2_ has a *K*_H_ value of 81.92, whereas [HyPropDBU]N(CN)_2_ shows a *K*_H_ value of 55.83. Among the six ILs, [HyPropDBU]NTf_2_ stands out as the most promising solvent for CO_2_ sorption, displaying the lowest *K*_H_ value at 30.80. The variations in anions paired with the same cations revealed a decreasing trend in *K*_H_ values as follows: NTf_2_ > N(CN)_2_ > SCN. Specifically, for the [HyPropDBU]^+^ cation paired with [NTf_2_] anion, the *K*_H_ value was 30.80, whereas N(CN)_2_ and SCN exhibited higher values of 55.83 and 71.84, respectively. Hence, the order of *K*_H_ value for the studied ILs across all composition ranges is: [HyPropDBU]NTf_2_ > [HyEtDBU]NTf_2_ > [HyPropDBU]N(CN)_2_ > [HyPropDBU]SCN > [HyEtDBU]N(CN)_2_ > [HyEtDBU]SCN.

## 4. Conclusion

In this work, a two-step procedure was successfully employed to synthesize six new DBU-based ILs. The study focused on characterizing their thermophysical properties, including density and thermal stability. The experimental results revealed a clear dependence of these properties on factors such as the linker chain length of the cation, the size of the anion, and pressure. Additionally, the analysis of physicochemical properties showed that molar mass had a significant influence on molar volume (*V*_m_), molecular volume (*V*), entropy (*S*°), potential energy (*U*_pot_) and free volume (*V*_f_). Among these, *V*_f_ was found to play a key role in determining the ILs’ capacity for CO_2_ absorption. Specifically, [HyPropDBU]NTf_2_, which had the largest *V*_f_, demonstrated the highest mole fraction of absorbed CO_2_, at 0.9573. This IL also exhibited the lowest Henry’s Law constant (*K*_H_), further confirming its superior CO_2_ absorption performance compared with the other five ILs studied. These results highlight [HyPropDBU]NTf_2_’s potential as a promising solvent for CO_2_ capture applications. DBU]-hydroxyl ILs should be considered in green chemistry applications owing to their potential use as solvents in CO_2_ capture. Their use in these applications can lead to reduced environmental footprint compared with traditional solvents. Future research should focus on investigating the CO_2_ desorption behaviour of these ILs to evaluate their recyclability and enhance their sustainability as absorbents for CO_2_ capture.

## Data Availability

The datasets supporting this article have been uploaded as part of the online supplementary material [55].
